# Looking beyond the heart in cardiac magnetic resonance imaging: an interesting case report of Takayasu arteritis presenting with left ventricular apical aneurysm

**DOI:** 10.1093/ehjcr/ytaf125

**Published:** 2025-03-14

**Authors:** Apratim Roy Choudhury, Jineesh Valakkada, Anoop Ayappan

**Affiliations:** Department of Imaging Sciences and Interventional Radiology, Sree Chitra Tirunal Institute for Medical Sciences and Technology (SCTIMST), Trivandrum, Kerala, India; Department of Imaging Sciences and Interventional Radiology, Sree Chitra Tirunal Institute for Medical Sciences and Technology (SCTIMST), Trivandrum, Kerala, India; Department of Imaging Sciences and Interventional Radiology, Sree Chitra Tirunal Institute for Medical Sciences and Technology (SCTIMST), Trivandrum, Kerala, India

**Keywords:** Case Report, LV aneurysm, Takayasu arteritis, Cardiac MRI

## Abstract

**Background:**

Takayasu arteritis (TA), a type of large vessel vasculitis involving the aorta and its major branches, is highly prevalent in Southeast Asian countries. Although cardiac involvement is rare, it is the most common cause of mortality in TA.

**Case summary:**

Here, we present a case of a 35-year-old woman, with no known previous co-morbidities, who presented with sudden onset acute chest pain and dyspnoea. Twelve-lead electrocardiogram revealed ST-elevation and positive troponin, leading to a clinical diagnosis of ST-elevation myocardial infarction. However, a coronary angiogram showed normal coronaries with an left ventricular (LV) apical outpouching. On table echocardiogram also showed an outpouching from the LV apex. Blood work revealed raised inflammatory markers. For further characterization of the outpouching, a cardiac magnetic resonance imaging (MRI) was done, which revealed an LV apical aneurysm along with ostial stenosis and wall enhancement of bilateral subclavian arteries. Further, the magnetic resonance angiogram showed ostial stenosis of bilateral common iliac origin. The extra-cardiac imaging features of aorto-arteritis, along with raised inflammatory markers led to the diagnosis of TA in this case.

**Discussion:**

This case report highlights the importance of looking beyond the heart in cardiac MRI. Ostial stenosis and enhancement of bilateral subclavian arteries in led to the imaging suspicion of TA. Further imaging of the entire arterial tree confirmed the diagnosis.

**Learning points**

- Cardiac involvement, albeit rare is the most common cause of mortality in Takayasu arteritis.
- Takayasu arteritis should be one of the differentials in young female patients presenting with myocardial infarction with normal coronary arteries.
- It is important to look beyond the heart in all the extra-cardiac structures in a cardiac magnetic resonance imaging scan.

## Introduction

Takayasu arteritis (TA) is a granulomatous immune-mediated large vessel vasculitis involving the aorta, its major branches and the pulmonary arteries. The disease commonly affects women in their second to fourth decade of life.^1^ The incidence of the disease varies geographically, with 1–2 cases per million in South East Asian countries.^2^ Cardiac involvement is a less-described clinical aspect of TA in the literature; however, it is the most common cause of mortality in TA.^3^ The most common cardiac manifestation in TA is aortic regurgitation (AR) due to valvular inflammation, followed by coronary arteritis and steno-occlusive coronary lesions.^3^ Over the years, multiple diagnostic criteria have been put forward for the diagnosis of TA. One commonly used criterion was the Sharma Criteria, where coronary artery involvement was considered a minor criterion.^4^ The latest criteria are the 2022 ACR EULAR Criteria, where ischaemia cardiac pain or angina is given +2 points.^5^ Here, we present a rare clinical manifestation of TA in the form of left ventricular (LV) apical aneurysm without any underlying coronary artery disease.

## Summary figure

**Figure:**
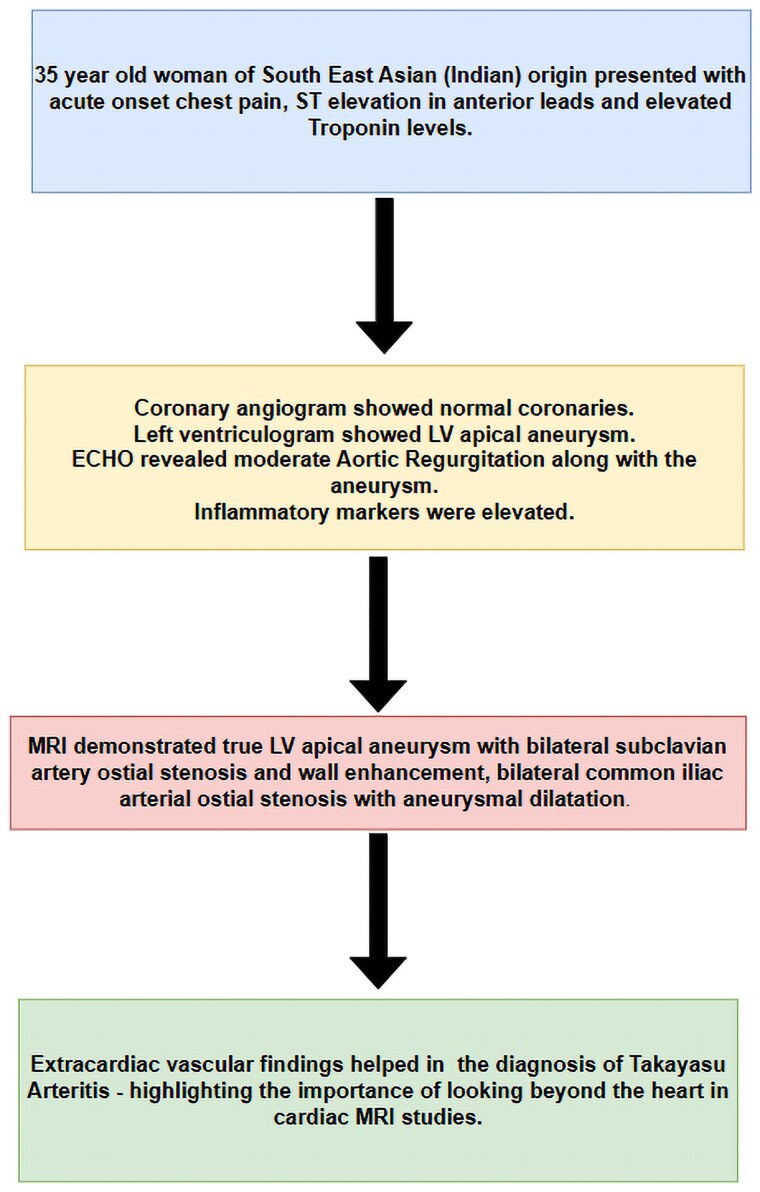


## Case presentation

A 35-year-old lady of Southeast Asian (Indian) origin presented to the emergency department with sudden onset retrosternal chest pain for the last 8 h, dyspnoea and palpitations. The symptomatology was sudden and was not precipitated by any exercise or exertion. She had no known co-morbidities or any significant medical/surgical history. On examination, she had sinus tachycardia (115 beats/min), blood pressure of 90/60 mmHg and jugular venous pressure elevated to the angle of the mandible. Precordial examination and chest auscultation revealed left ventricular third heart sound and bilateral basal lung crackles. Electrocardiogram (ECG) showed ST-segment elevation in the antero-inferior leads with elevated Troponin I level of 1.2 ng/mL. Chest X-ray (CXR) revealed mild cardiomegaly with a cardio-thoracic ratio of 0.62 and Grade I pulmonary venous hypertension.

**Figure 1:**
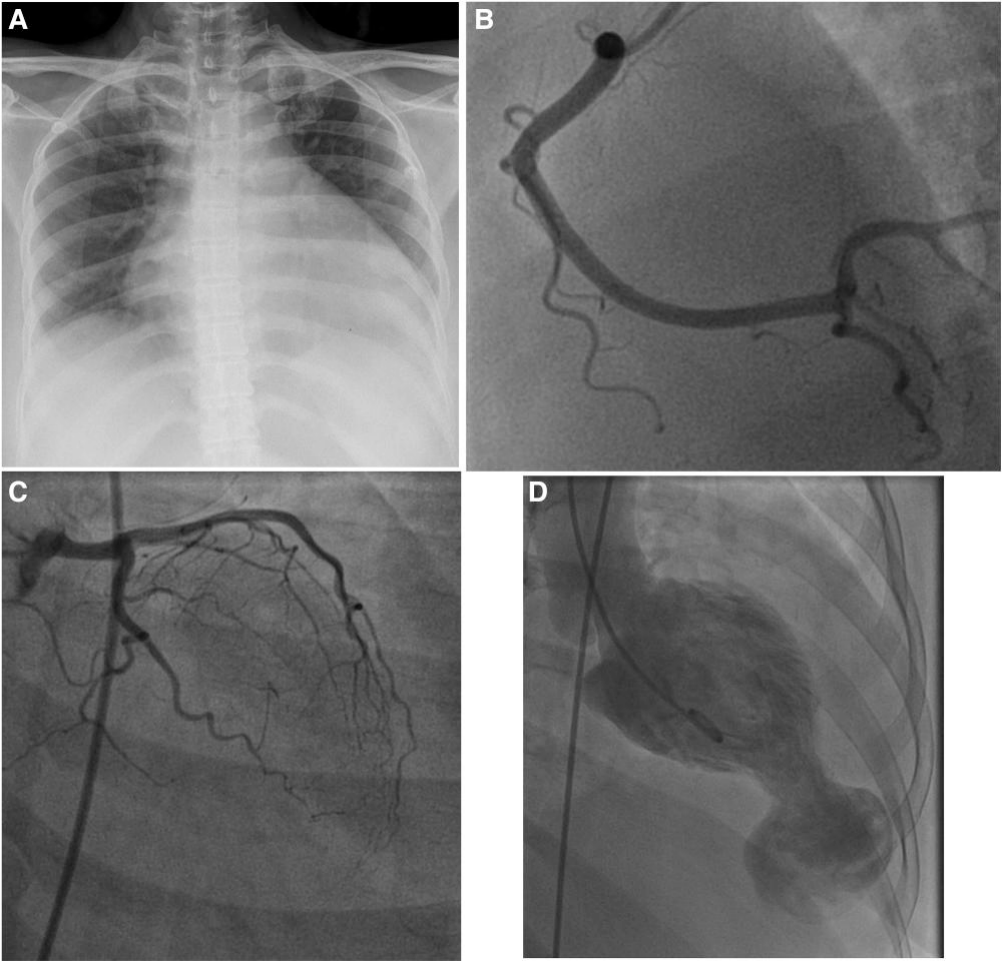
CXR (*A*) frontal projection showing cardiomegaly. A catheter coronary angiogram shows normal coronaries (*B* and *C*). A diagnostic ventriculogram (*D*) image shows a large left ventricular apical aneurysm.

Because of the provisional diagnosis of type I acute myocardial infarction, she was immediately shifted to primary percutaneous coronary intervention. However, the angiogram revealed normal coronaries (*Figure 1B* and *C*), and a left ventriculogram revealed a large apical aneurysmal outpouching (*Figure 1D*). On table echocardiogram confirmed the ventriculogram findings of LV apical outpouching with preserved thickness and contractility of the basal and mid-ventricular segments along with moderate AR, ejection fraction of 64% and mild pericardial effusion.

Labs showed an elevation of N-terminal pro-brain natriuretic peptide (2240 pg/mL). Other blood work revealed mild anaemia (haemoglobin of 8.7 gm/dL), elevated erythrocyte sedimentation rate (ESR) of 82 mm/first hour and elevated C-reactive protein (88.92 mg/L) with normal white blood cell count, renal, and liver function tests. Symptomatic LV apical aneurysm with normal coronaries and elevated serum inflammatory markers led to a diagnostic dilemma. The possible differentials were Takotsubo cardiomyopathy, LV apical aneurysm secondary to remote embolic infarct, and a rare possibility of Chaga’s disease.

After the multi-disciplinary team (MDT) discussion, a cardiac magnetic resonance imaging (MRI) was planned to characterize the outpouching and look for any evidence of myocardial ischaemia from microvascular aetiology. The patient was started with 75 mg of a once daily dose of Aspirin, 25 mg of metoprolol succinate, and a daily 10 mg dose of enalapril.

**Figure 2:**
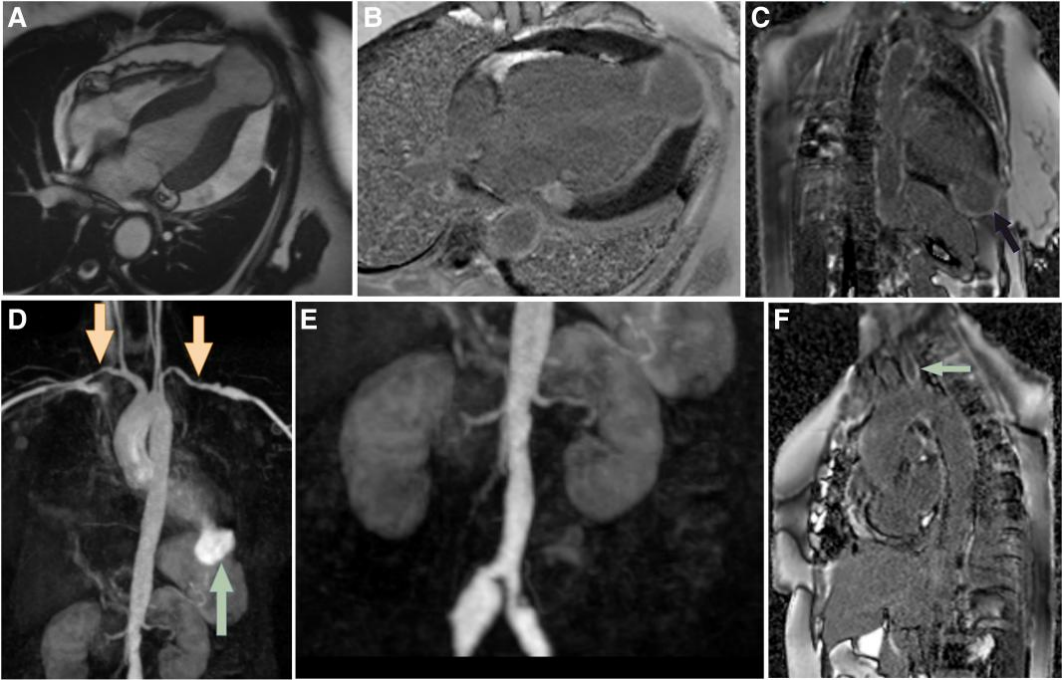
Balanced steady state free precision (*A*) four-chamber view of the heart showing an aneurysmal outpouching from the left ventricular apex. Late gadolinium enhancement (LGE) in four chambers (*B*) and two-chamber views (*C*) image showing intense delayed enhancement. Coronal maximum intensity projection (MIP) MR angiogram (*D*) showing bilateral multifocal subclavian stenosis (yellow arrows). The LV aneurysm is marked by the green arrow. Coronal MIP MR angiogram (*E*) shows bilateral common iliac arteries (CIA) stenosis with associated aneurysmal dilatation of right CIA and ectatic left CIA. Post-contrast delayed image (*F*) showing circumferential late gadolinium enhancement along the most proximal segment of the left subclavian artery.

The MRI revealed a 3.6 × 2.5 cm aneurysmal outpouching in the apex of the left ventricle (*Figure 2A*). The neck was relatively narrow, measuring 1.3 cm. Thinned-out myocardium was seen lining the aneurysmal outpouching with rhythmic dyskinetic contractility and intra-aneurysmal swirling of blood. Delayed late gadolinium enhanced (LGE) MRI showed intense peri-aneurysmal enhancement. (*Figure 2B* and *C*). There were no significant peri-aneurysmal inflammatory changes. In view of the dyskinetic contraction of the sac, swirling of blood and absence of adjacent inflammatory changes, a diagnosis of a true aneurysm was considered. There were no other significant cardiac findings.

However, there were multiple extra-cardiac findings. There were multifocal areas of severe stenosis in the bilateral subclavian arteries (*Figure 2D*). The ostia of bilateral common iliac arteries were stenosed, with post-stenotic aneurysmal dilatation/ectasia (*Figure 2E*). In addition, there was significant stenosis of the left renal artery. Delayed LGE images showed enhancement of the left common carotid artery wall at its ostia (*Figure 2F*), and the bilateral common carotids were diffusely narrowed.

Based on raised inflammatory markers and MRI signs of vasculitis, a diagnosis of TA was reached. The patient was started on appropriate medical management of steroids and anti-inflammatory drugs. After the inflammatory markers came down to normal levels, the patient ultimately underwent aneurysmectomy and surgical reconstruction (Dor’s Procedure) with good clinical outcome.

## Discussion

This case is a rare manifestation of TA and demonstrates the importance of extra-cardiac findings in cardiac MRI scans.

Takayasu arteritis, a type of large vessel vasculitis, usually presents in the age group of 20–40 years, commoner in females with a high prevalence rate in Southeast Asian countries like India and Japan.^2^ The hallmark clinical features of TA are upper limb claudication, headache, hypertension, asymmetric limb blood pressure, and other constitutional symptoms.^6^ Cardiac involvement is a common but less recognized manifestation of TA. It is the most common cause of death in TA.^3^

Takayasu arteritis can involve any structure of the heart. The most common manifestation is AR, which is caused by annular dilatation caused by aortitis.

Left ventricular aneurysm in TA can happen by two pathways. One is coronary arteritis, in which the myocardium supplied can go into infarction and subsequently undergo aneurysm/pseudo-aneurysm formation.^7^ The second pathway is primary myocarditis, causing myocardial necrosis and aneurysmal outpouching.^8^ There can also be an overlap in the two pathways. In our case, because of relatively normal epicardial coronaries, we suspect primary myocarditis to be the cause. However, the possibility of microvascular ischaemia exists.

Another aspect of this case was the apical aneurysm. Left ventricular apex pseudo vs. true aneurysm is a diagnostic challenge, and MRI is a valuable modality for diagnosis.^9^ True aneurysms are focal outpouching, having all three layers of endocardium, myocardium, and epicardium. It is usually wide-necked (defined as neck diameter >50% of the maximum transverse diameter of the aneurysm) and is common in the apical, anterior, and anterolateral segments of the myocardium. On the contrary, a pseudo-aneurysm is basically a contained rupture. It has no endomyocardial lining, is just contained by the pericardium, has a narrow neck, and is more common on the lateral and inferior walls of the LV. The main point in differentiating these two entities—is the demonstration of dyskinetic contractility of the aneurysm wall. It indicates myocardium lining the aneurysm sac and thus a true aneurysm.^10^

Cardiac MRI, in light of clinical findings, is an indispensable tool to rule out other differentials. Takotsubo cardiomyopathy—the so-called stress-induced cardiomyopathy also presents with classical apical outpouching. It is commonly seen in postmenopausal women after life-altering physical or emotional stress. The sudden emotional trigger activates the limbic system, leading to a sympathetic overdrive and stimulating the adrenergic receptors. The beta receptors in the base of the heart go for a hyper-contractile state, while the stimulation of the alpha receptors leads to apical relaxation. Thus, Takotsubo has a hyper-contractile base of the heart with transient apical ballooning.^11^ The Mayo diagnostic criteria are commonly used to diagnose Takotsubo, and the absence of pheochromocytoma or myocarditis is mandatory. In our case, the apical ballooning was not transient; the base of the heart was not hyper-contractile with intense delayed enhancement, indicating inflammation. All these factors, in our case, were against the diagnosis of Takotsubo.

A second differential of LV apical aneurysm is chronic Chaga’s cardiomyopathy.^12^ Chronic Chaga’s cardiomyopathy commonly presents with cardiomegaly, bundle branch blocks, LV systolic dysfunction, and LV apical aneurysm. It is an important differential in places endemic for *Trypanosoma cruzi* (*T. cruzi*). Our area is non-endemic, and our patient has no significant travel history. Her serology for *T. cruzi* was also negative; hence, it was not considered.

The third possibility was a remote embolic infarct, which might have caused an apical infarction and secondary aneurysm formation. It was a possible diagnosis, but the patient never had any previous episode of chest pain and thus was considered a less likely possibility. The ECG also revealed no pathological q waves along the anterolateral leads.

Besides ruling out differentials, the importance of MRI, in this case, lies in demonstrating the extra-cardiac findings. Multivessel stenosis and aneurysmal dilatation are the hallmarks of TA. Both luminal stenosis and inflammatory wall thickening are depicted by MRI. Thus, MRI has now replaced conventional angiography as the ‘gold standard’ imaging modality for TA diagnosis in the recent EULAR imaging recommendations. Radiologists need to look beyond the heart while evaluating cardiac MRIs.

## Conclusion

To conclude, TA, a common vasculitis in the Asian population, can cause coronary arteritis, myocarditis, AR, and other cardiac abnormalities. This case demonstrates an LV apical aneurysm, a rare cardiac manifestation of TA without any concomitant epicardial coronary artery disease.

## Supplementary Material

ytaf125_Supplementary_Data

## Data Availability

The data underlying this article are not publicly available due to patient privacy concerns, but can be accessed upon reasonable request to the corresponding author with appropriate ethics approval.

## Lead author biography

Dr Apratim is working as a Senior Department in the Radiology Department of SCTIMST, Trivandrum. He has keen interest in various aspects of cardiac CT and cardiac MRI—especially imaging in congenital heart diseases.

## Notes

**Consent:** The authors confirm that written consent for submission and publication of this case, including images and associated text, has been obtained from the patient in line with COPE guidance.

**Funding:** None declared.
